# Heterogeneous trajectories of kinesiophobia and their effects on rehabilitation outcomes after total knee arthroplasty: a prospective cohort study

**DOI:** 10.1186/s13018-023-03881-8

**Published:** 2023-06-23

**Authors:** Zeping Yan, Yu Wu, Mengqi Liu, Xiaoli Wang, Jiurui Wang, Zhiwei Wang, Shicai Wu, Xiaorong Luan

**Affiliations:** 1 School of Nursing and Rehabilitation, Cheeloo College of Medicine, Shandong University, Jinan, China; 2 University of Health and Rehabilitation Sciences, Qingdao, China; 3 Beijing Bo’ai Hospital, China Rehabilitation Research Center, Beijing, China; 4 School of Nursing and Rehabilitation, Qilu Hospital, Shandong University, Jinan, China

**Keywords:** Kinesiophobia, Rehabilitation, Trajectory analysis, Total knee arthroplasty

## Abstract

**Background:**

Kinesiophobia is one of the most common and aversive psychological phenomena among patients after total knee arthroplasty (TKA). This study aimed to identify trajectories of kinesiophobia, examine factors distinguishing these trajectories, and clarify the association between trajectories of kinesiophobia and rehabilitation outcomes.

**Methods:**

In this prospective cohort study, the patients who underwent TKA were recruited between December 2021 and April 2022 from three orthopedic wards of a tertiary hospital in China. Kinesiophobia was measured using the Tampa Scale for Kinesiophobia at baseline (*T*0), and then at 1 month (*T*1) and 3 months (*T*2) after TKA to perform latent class growth analysis. Meanwhile, rehabilitation outcomes were assessed at 3 months after TKA, using the Kessler Psychological Distress Scale, the Hospital for Special Surgery-Knee Scale, Barthel Index, and the Impact on Participation and Autonomy questionnaire.

**Results:**

The four kinesiophobia trajectories identified were as follows: low stable group (*n* = 120), rapid recovering group (*n* = 31), slow recovering group (*n* = 48), and stable moderate group (*n* = 58). Body mass index, employment status, heart disease, and pain degree significantly predicted trajectory groups (all *p* < 0.05). Analysis of variance revealed significant differences between the four kinesiophobia trajectories concerning all rehabilitation outcomes, except for the activities of daily living.

**Conclusion:**

Distinct kinesiophobia trajectories were identified, and nurses should assess the kinesiophobia of patients after TKA in the early phase. Patients in the slow recovering group are worthy of a specific focus because of their poor recovery after undergoing TKA. As important sources of psychosocial care, nurses need to customize psychological interventions for patients after TKA depending on each kinesiophobia trajectory.

## Introduction

Knee osteoarthritis is the most prevalent osteoarticular condition and a major contributor to years lived with disability worldwide, damaging all joint anatomical structures [1]. Meanwhile, total knee arthroplasty (TKA) is a highly successful established technology, and the only definitive therapy available is recommended to treat end-stage knee osteoarthritis [2]. In China, almost 400,000 TKA were performed in 2019, and it will continue to increase [3].

Although outcomes after TKA have improved on average, up to 20% of patients suffered from prolonged pain, joint stiffness, and lower satisfaction [4, 5]. Pieces of evidence involving patients who underwent arthroplasty have highlighted that kinesiophobia is a crucial factor contributing to the success of rehabilitation in various health conditions, particularly orthopedic surgery [6–8]. Kinesiophobia refers to an excessive and irrational psychological phenomenon in which individuals have the debilitating fear for rehabilitation exercise or activity affected by fear of re-injury or pain experience, which can prolong or hinder functional improvement [6]. Based on the fear-avoidance model, kinesiophobia may lead to psychological troubles and subsequent poor physical performance [9, 10]. In addition, cross-sectional studies indicated that kinesiophobia was associated with poor physical function and high psychological disability, which were in favor of the fear-avoidance model [7, 11].

Herein, the wide-reaching implications of kinesiophobia for physical and psychological rehabilitation have been discussed using variable-centered methods, which have not considered population-level heterogeneity. That is, most empirical studies focusing on kinesiophobia have assessed the independent effects of the mean scores of kinesiophobia on rehabilitation outcomes and have been assessed at a single time point [12, 13]. This approach neglects the course of kinesiophobia over time. Latent class growth analysis (LCGA) can capture information about interindividual differences in the change of kinesiophobia over time and identify participants with similar kinesiophobia trajectories [14]. Liang has identified three distinct trajectories of kinesiophobia in a sample of participants with chronic obstructive pulmonary disease [15]. The three groups were named low kinesiophobia, medium kinesiophobia, and high kinesiophobia group, respectively. However, the trajectories of kinesiophobia among patients undergoing TKA have not been studied yet. Given that kinesiophobia is a dynamic and individualized adjustment process, exploring different trajectories of kinesiophobia might provide further insights into the complex association of kinesiophobia with rehabilitation outcomes.

As proposed by the World Health Organization in 2001, the International Classification of Functioning, Disability, and Health (ICF) has been the basis and guideline in the field of rehabilitation research, which covers different aspects of body functions (including mental functions), activity, and participation [16]. Social participation is viewed as the most relevant outcome in rehabilitation [17]. ICF defines participation as “connecting with people and the community,” thus representing the social perspective of functioning, whereas the activity is deemed to function at the level of the individual [17]. However, studies aiming to examine the effect of kinesiophobia on rehabilitation outcomes only focused on physical and psychological function and ignored social participation. Therefore, it is necessary to conduct a comprehensive and integrated evaluation of the influence of kinesiophobia on rehabilitation outcomes for patients after TKA based on the ICF framework. In this study, we examined four rehabilitation outcomes after the trajectory period ended: psychological distress, knee function, activities of daily living, and social participation.

In summary, the existence of distinct kinesiophobia trajectories remains unexplored; furthermore, the longitudinal relationships of kinesiophobia trajectories with rehabilitation outcomes have not been studied. Given these knowledge gaps, this study aimed to: (1) identify the kinesiophobia trajectories of patients after TKA using LCGA; (2) determine factors at baseline that predict these trajectories; and (3) verify whether different kinesiophobia trajectories were associated with rehabilitation outcomes.

## Methods

### Ethical considerations

The study was approved by the Ethics Committee of the University (Reference Number: 2021-R-031) and complied with the principles of the Declaration of Helsinki. Informed consent was obtained from the participants.

### Study design and participants

A prospective cohort study was conducted. Patients undergoing primary TKA were recruited from three orthopedic wards of a tertiary hospital by convenience sampling. The recruitment period was from December 2021 to April 2022. Demographic and comorbidity characteristics were collected at baseline (*T*0). The baseline was measured on the day when the patients admitted to hospital to receive TKA. In addition, patients completed a survey questionnaire about kinesiophobia at *T*0 and during two follow-up periods at 1 month (*T*1) and 3 months (*T*2) after TKA. Patients’ rehabilitation outcomes were obtained at 3 months after TKA (*T*2). Patients’ inclusion criteria were as follows: (1) aged more than 45 years; (2) received unilateral TKA; and (3) speak and understand Chinese without communication disorder. Exclusion criteria were as follows: (1) undergoing TKA for an indication other than knee osteoarthritis and (2) having a history of TKA. A total of 300 patients were screened and completed baseline questionnaires at *T*0. Meanwhile, 19 and 11 patients were lost to follow-up at *T*1 and *T*2, respectively. Moreover, four patients experienced adverse events and nine patients received contralateral TKA were excluded. The final sample consisted of 257 patients for data analysis (85.67%).

### Measures

The baseline characteristics of demographic and comorbidity included age, gender, body mass index (BMI), educational level, residence, employment status, marital status, smoking, surgical site, heart disease, diabetes, hypertension, and pain degree. Pain degree was assessed by an 11-point numerical rating scale, which could be divided into no (0), mild (1–3), moderate (4–6), and severe pain (7–10) [18].

#### Kinesiophobia

The shortened version of the Tampa Scale for Kinesiophobia (TSK) was employed to measure kinesiophobia [19]. It was an 11-item scale, and each item was rated on a 4-point scale, ranging from 1 (strongly disagree) to 4 (strongly agree). Furthermore, scores on the TSK-11 ranged from 11 to 44, with higher scores indicating greater perceived levels of kinesiophobia.

#### Rehabilitation outcomes

The psychological distress was assessed by the Kessler Psychological Distress Scale-10 items (K-10) [20]. Each item with a five-level response was scored from 1 (none of the time) to 5 (all time). The total score ranged from 10 to 50, with higher scores indicating greater levels of psychological distress.

The Hospital for Special Surgery-Knee Scale (HSS-KS) was used to evaluate knee function [21]. HSS-KS was a valid and reliable assessment tool, including pain, function, range of motion, myodynamia, flexion deformity, stability, and subtraction items. In addition, the total HSS-KS score was 100 points, with higher scores indicating better knee function.

Barthel Index (BI) was composed of 10 items to assess activities of daily living [22]. The score ranged from 0 (totally dependent) to 100 (independent). Previous evidence also showed that BI could effectively predicate activities of daily living among the population after TKA [23].

The Chinese version of the Impact on Participation and Autonomy questionnaire (IPA) consisted of 25 items intended to measure social participation [24]. Each item was answered on a 5-point scale from 0 (very good) to 4 (very poor). The lower the total score, the better social participation.

### Statistical analysis

To identify heterogeneity in the patterns of the kinesiophobia subgroups among patients after TKA, growth mixture modeling (GMM) and LCGA were performed using Mplus, version 7.4. In our model, time was modeled discretely because we only conducted three surveys on the TSK. A quadratic growth model requires a minimum of four time points to estimate all of its parameters [25]. A combination of fit indices and substantive interpretation was recommended for determining the appropriate number of trajectory groups [26]. The lower values of the Akaike information criterion (AIC), Bayesian information criterion (BIC), and adjusted Bayesian information criterion (aBIC) indicated a better-fitting model [14, 27]. Entropy ranged from 0 to 1, and the value of entropy greater than 0.80 was graded as adequate classification precision [28]. Meanwhile, the Vuong–Lo–Mendell–Rubin likelihood ratio test (VLMR) and bootstrapped likelihood ratio test (BLRT) were used to compare the improvement between neighboring LCGA models [26]. Lastly, when choosing the numbers of subgroups, it is important to ensure that each subgroup has a sample size of no less than 5% of the total population [29]. The final kinesiophobia model was selected by comparing the optimal fitting model indices between the LCGA and GMM approaches.

Differences between trajectories and baseline characteristics were evaluated by the Chi-squared test or Fisher’s exact test. All variables with *p *values < 0.1 were chosen as independent variables and further analyzed by multinomial logistic regression. Analysis of variance and post hoc tests were conducted using *R* software version 4.1.1 to examine the longitudinal relationship between each kinesiophobia trajectory and rehabilitation outcomes among patients after TKA. Furthermore, the adjusted *p* value was calculated using Bonferroni correction to correct the risk of type 1 error.

## Results

### Study sample

The patients included in this study were 170 females (66.1%) and 87 males (33.9%). The mean age of the patients was 63.60 ± 7.52 years. Most of the patients reported BMI ≥ 25 kg/m^2^ (72.4%), were married (93.4%), received right TKA (53.7%), and reported moderate pain (60.7%). Meanwhile, the number of patients with heart disease, diabetes, and hypertension was 27 (10.5%), 21 (8.2%), and 100 (38.9%), respectively.

### Identifying trajectories of kinesiophobia

We compared the information-based fit indices of LCGA and GMM models from one to five latent trajectories, as shown in Table 1. In the LCGA model, the VLMR tests for the five- and three-trajectory models were not significant, thereby indicating that the five-trajectory model did not outperform the four-trajectory model, and the three-trajectory model was poorer than the two-trajectory model. Meanwhile, the AIC, BIC, and aBIC values of the four-trajectory model were lower than the two-trajectory model. The entropy value of the four-trajectory model was 0.874, thus providing a clear classification. In the GMM model, the VLMR tests for the five-trajectory models were not significant, which supported the four-trajectory model. However, one of the classes in the four-trajectory had inadequate sample size. Finally, based on a comparison of the model fit indices between LCGA and GMM approaches, the four-trajectory LCGA model was concluded to be the optimal model for kinesiophobia.

**Table 1:** Model fit indices for one to five trajectories of kinesiophobia

Model	Number of trajectories	AIC	BIC	aBIC	Entropy	VLMR *p *value	BLRT *p *value	Latent trajectory proportions (%)
LCGM	1	5204.756	5215.403	5205.892	–	–	–	100
	2	4790.742	4812.036	4793.015	0.900	< 0.001	< 0.001	65.37/34.63
	3	4704.614	4736.556	4708.023	0.842	0.1037	< 0.001	22.18/48.64/29.18
	**4**	**4620.420**	**4663.009**	**4624.965**	**0.874**	**0.0178**	** < 0.001**	**46.69/12.06/18.68/22.57**
	5	4560.634	4613.871	4566.316	0.872	0.7535	< 0.001	18.29/12.06/39.30/4.28/26.07
GMM	1	4664.577	4692.970	4667.607	–	–	–	100
	2	4699.280	4689.320	4604.446	0.870	< 0.001	< 0.001	33.21/66.79
	3	4572.220	4621.907	4577.522	0.905	0.0022	< 0.001	2.87/33.33/63.80
	4	4518.796	4579.130	4525.235	0.911	0.0115	< 0.001	9.20/4.74/24.87/61.19
	5	4504.727	4575.709	4512.303	0.854	0.0611	< 0.001	35.76/21.35/29.98/8.27/4.64

The VLMR and BLRT of the 4-trajectory model in LCGM were all statistically significant (*p* < 0.05) and each subgroup had a sample size of more than 5% of the total population

*AIC* Akaike Information Criterion, *BIC* Bayesian Information Criterion, *aBIC* adjusted Bayesian Information Criterion, *VLMR* Vuong–Lo–Mendel–Rubin likelihood ratio test, *BLRT* Bootstrapped likelihood ratio test

**Fig. 1:**
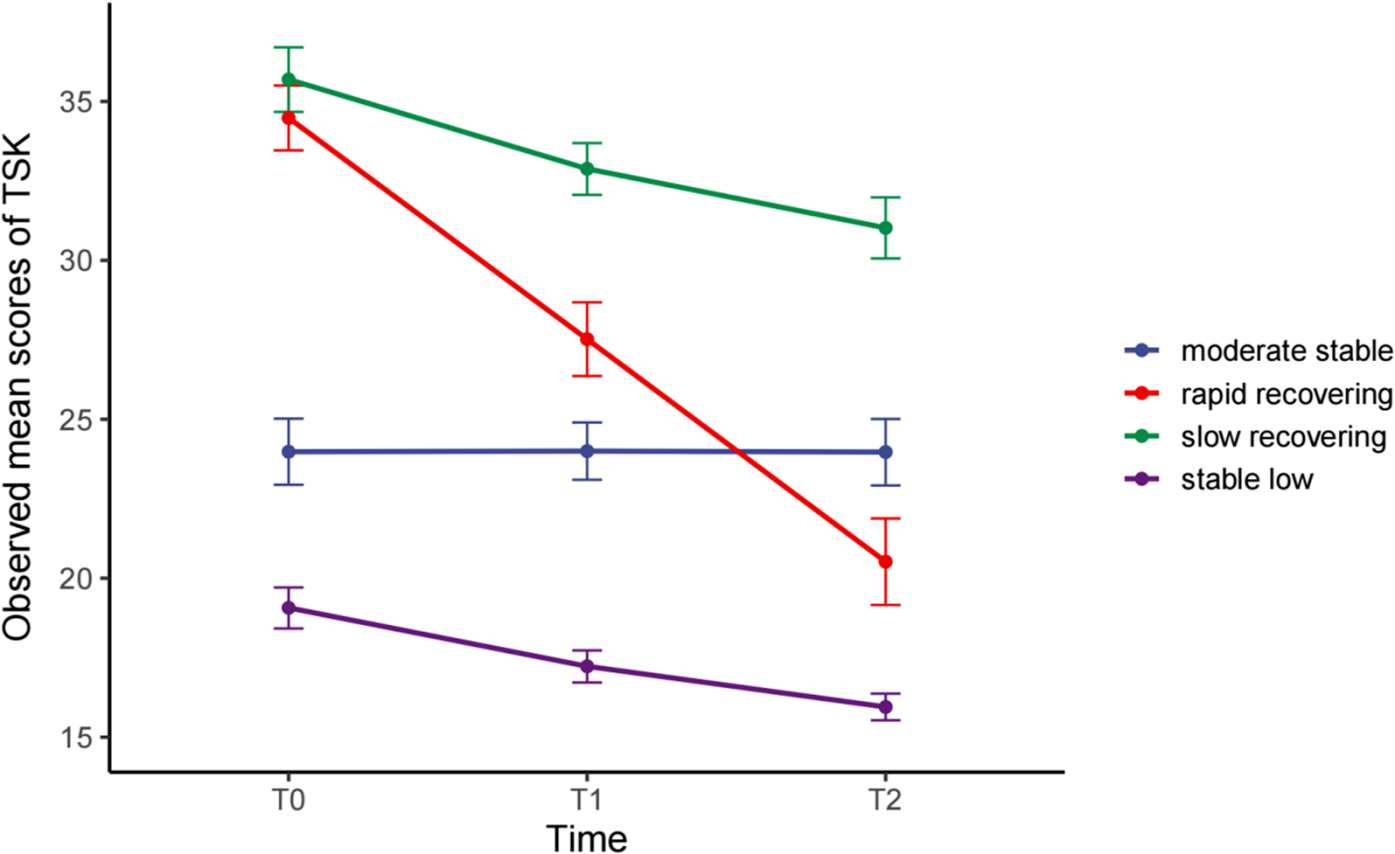
Longitudinal trajectories of kinesiophobia. The x-axis represented the follow-up time and the y-axis showed the observed mean scores of TSK at each time point. Results are displayed as observed mean scores of TSK with 95% confidence interval

Figure 1 provides a graphical representation of the four trajectories of kinesiophobia. Accounting for the largest proportion of patients (*n* = 120, 46.7%), trajectory 1 was characterized by a relatively stable lowest value of TSK at 3-time points, which was named the low stable group. A total of 31 patients (12.1%) in trajectory 2 initially reported severe kinesiophobia with a sharp and rapid recovery in their kinesiophobia levels over time, which was named the rapid recovering group. Meanwhile, trajectory 3, with a similar initial score to the rapid recovering group in the T0 but exhibited a slow decline of TSK over time, was named the slow recovering group (*n* = 48, 18.7%). Trajectory 4 was named the moderate stable group because its patients reported consistently moderate levels of TSK over time, and 22.6% of patients (*n* = 58) belonged to this trajectory.

### Factors associated with kinesiophobia trajectories

**Fig. 2:**
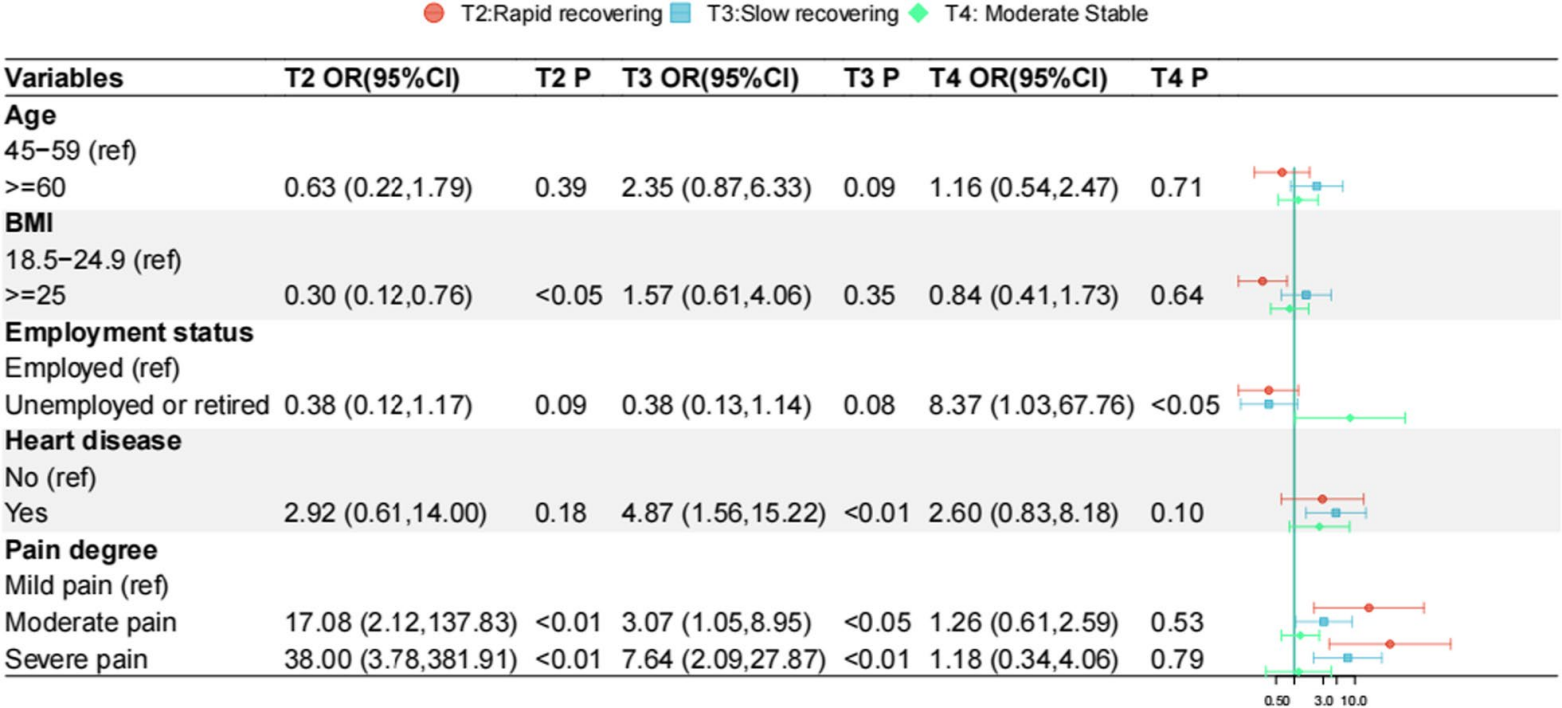
Differences in baseline characteristics across kinesiophobia trajectories. *OR* Odds ratio, *CI* Confidence interval, Low stable group was the reference category

Univariate analysis revealed that significant factors associated with different trajectories were age (*χ*^2^ = 10.903, *p* < 0.05), BMI (*χ*^*2*^ = 8.935, *p* < 0.05), employment status (*χ*^2^ = 22.608, *p* < 0.001), and heart disease (*χ*^2^ = 9.870, *p* < 0.05) (Table 2). Multinomial logistic regression analysis was conducted to get the variables’ influence to a further extent with the low stable group as the reference group (Fig. 2). In particular, patients falling into the rapid recovering group were more likely to report a BMI of 18.5–24.9 and moderate-to-severe pain compared with the low stable group. Similarly, the slow recovering group was significantly associated with moderate-to-severe pain. Meanwhile, those with heart disease were more likely to belong to the slow recovering group rather than the low stable group compared with patients without heart disease. In addition, patients who were unemployed or retired were underrepresented in the low stable group, while they were more represented in the moderate stable group.

**Table 2:** Comparison of baseline characteristics among different kinesiophobia trajectories

Characteristics	Overall (*N* = 257)	Kinesiophobia trajectories				*Χ*^2^	*p*
		Low stable (*N* = 120)	Rapid recovering (*N* = 31)	Slow recovering (*N* = 48)	Moderate stable (*N* = 58)		
Age						10.903	0.012
45–59	84 (32.7%)	44 (36.7%)	16 (51.6%)	10 (20.8%)	14 (24.1%)		
≥ 60	173 (67.3%)	76 (63.3%)	15 (48.4%)	38 (79.2%)	44 (75.9%)		
Gender						2.750	0.432
Male	87 (33.9%)	37 (30.8%)	14 (45.2%)	18 (37.5%)	18 (31.0%)		
Female	170 (66.1%)	83 (69.2%)	17 (54.8%)	30 (62.5%)	40 (69.0%)		
Body mass index (kg/m^2^)						8.935	0.030
18.5–24.9	71 (27.6%)	33 (27.5%)	14 (45.2%)	7 (14.6%)	17 (29.3%)		
≥ 25	186 (72.4%)	87 (72.5%)	17 (54.8%)	41 (85.4%)	41 (70.7%)		
Education						1.469	0.676
Middle school or lower	226 (87.9%)	104 (86.7%)	26 (83.9%)	43 (89.6%)	53 (91.4%)		
High school or higher	31 (12.1%)	16 (13.3%)	5 (16.1%)	5 (10.4%)	5 (8.6%)		
Residence						6.136	0.105
Urban	96 (37.4%)	47 (39.2%)	16 (51.6%)	18 (37.5%)	15 (25.9%)		
Rural	161 (62.6%)	73 (60.8%)	15 (48.4%)	30 (62.5%)	43 (74.1%)		
Employment status						22.608	< 0.001
Employed	40 (15.6%)	17 (14.2%)	12 (38.7%)	10 (20.8%)	1 (1.7%)		
Unemployed or retired	217 (84.4%)	103 (85.8%)	19 (61.3%)	38 (79.2%)	57 (98.3%)		
Marital status						0.757	0.888
Married	240 (93.4%)	112 (93.3%)	30 (96.8%)	44 (91.7%)	54 (93.1%)		
Divorced or widowed	17 (6.6%)	8 (6.7%)	1 (3.2%)	4 (8.3%)	4 (6.9%)		
Smoking	44 (17.1%)	18 (15.0%)	6 (19.4%)	8 (16.7%)	12 (20.7%)	1.017	0.797
Surgical site						4.345	0.227
Left	119 (46.3%)	61 (50.8%)	14 (45.2%)	16 (33.3%)	28 (48.3%)		
Right	138 (53.7%)	59 (49.2%)	17 (54.8%)	32 (66.7%)	30 (51.7%)		
Heart disease	27 (10.5%)	6 (5.00%)	3 (9.7%)	10 (20.8%)	8 (13.8%)	9.870	0.016
Diabetes	21 (8.2%)	6 (5.00%)	5 (16.1%)	3 (6.3%)	7 (12.1%)	5.590	0.115
Hypertension	100 (38.9%)	50 (41.7%)	8 (25.8%)	21 (43.8%)	21 (36.2%)	3.274	0.351
Pain degree						27.150	< 0.001
Mild pain	64 (24.9%)	41 (34.2%)	1 (3.2%)	5 (10.4%)	17 (29.3%)		
Moderate pain	156 (60.7%)	67 (55.8%)	23 (74.2%)	30 (63.5%)	36 (62.1%)		
Severe pain	37 (14.4%)	12 (10.0%)	7 (22.6%)	13 (27.1%)	5 (8.6%)

### Relationship between kinesiophobia trajectories and rehabilitation outcomes

**Fig. 3:**
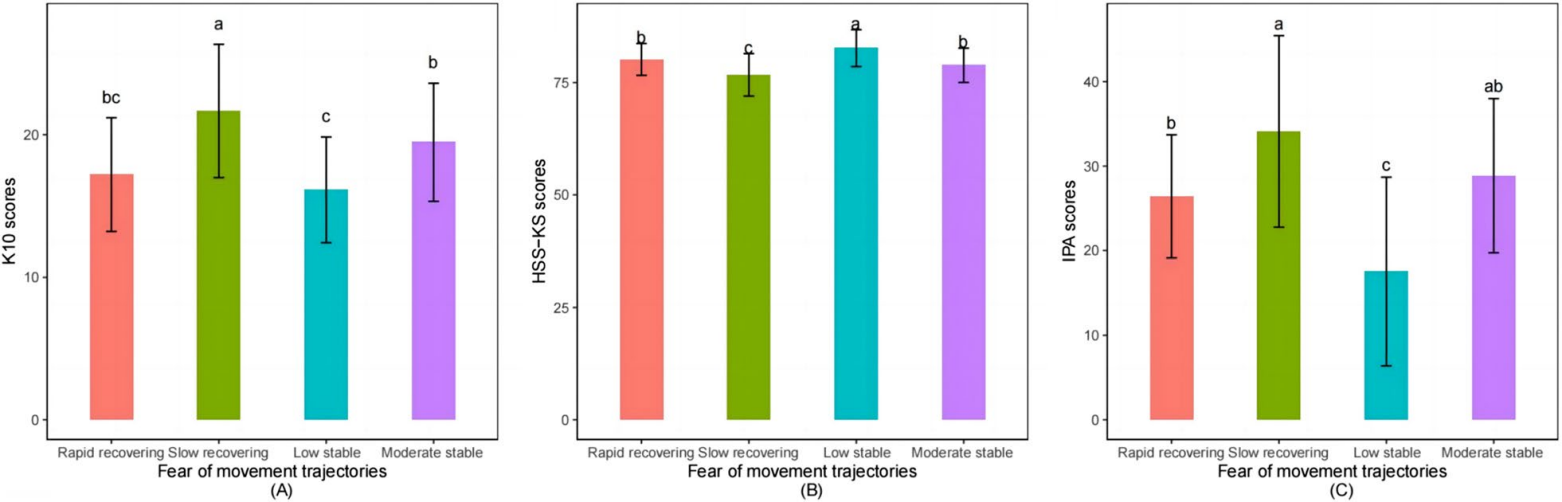
Post hoc analysis of rehabilitation outcomes differences among the subgroups of kinesiophobia trajectories. The means for **A** K10, **B** HSS-KS, and **C** IPA were presented by the bar plot. The lines represented the standard deviation. Different letters indicate significant differences according to Bonferroni post hoc test and subgroups of trajectories sharing the same letter were not significantly different

Differences in rehabilitation outcomes among kinesiophobia trajectories are detailed in Table 3 and Fig. 3. Analysis of variance showed a significant difference with respect to K-10 (*F* = 24.71, *p* < 0.001), HSS-KS (*F* = 27.87, *p* < 0.001), and IPA (*F* = 35.51, *p* < 0.001). However, no significant difference was observed between the kinesiophobia trajectories and BI (*F* = 0.80, *p* > 0.05). The results obtained from the post hoc tests indicated that the patients in the slow recovering group received significantly higher scores on K-10 than the other three groups, thereby suggesting that patients in the slow recovering group had worse psychological distress. Meanwhile, the slow recovering group had lower HSS-KS scores compared to the other groups, thus indicating worse knee function in the slow recovering group. Significant differences in IPA scores revealed that the low stable group had higher scores than other groups. That is, patients belonging to the low stable group have a high level of social participation at 3 months after TKA.

**Table 3:** Rehabilitation outcomes differ across different kinesiophobia trajectories

Rehabilitation outcomes	Trajectory 1 M (SD)	Trajectory 2 M (SD)	Trajectory 3 M (SD)	Trajectory 4 M (SD)	*F*
K-10	16.13 (3.72)	17.19 (4.00)	21.67 (4.68)	19.47 (4.15)	24.71^***^
HSS-KS	82.68 (4.10)	80.16 (3.54)	76.73 (4.72)	78.86 (3.82)	27.87^***^
BI	93.58 (3.84)	92.74 (3.12)	93.54 (14.73)	91.90 (4.06)	0.80
IPA	17.53 (11.16)	26.42 (7.28)	34.10 (11.35)	28.86 (9.14)	35.51^***^

*K-10* Kessler Psychological Distress Scale-10 items, *HSS-KS* The Hospital for Special Surgery-Knee Scale, *BI* Barthel Index, *IPA* Impact on Participation and Autonomy questionnaire, *Trajectory 1* Low stable, *Trajectory 2* Rapid recovering, *Trajectory 3* Slow recovering, *Trajectory 4* Moderate stable

^***^p < 0.001

## Discussion

This is the first known study to reveal distinct kinesiophobia trajectories among patients after TKA by LCGA, and some important results were obtained. Overall kinesiophobia trajectories after TKA were largely classified into four groups: low stable group, rapid recovering group, slow recovering group, and moderate stable group. In addition, this study gave particular interest in BMI, employment status, heart disease, and pain degree differentiated by these kinesiophobia trajectories. Our results add to knowledge concerning kinesiophobia, pointing that each kinesiophobia trajectory exhibited different associations with rehabilitation outcomes.

Different from the research carried out by Liang [15], kinesiophobia among patients undergoing TKA showed four trajectories. The low stable group and moderate stable group in our study were very similar to the low kinesiophobia group and medium kinesiophobia group in Liang’s study. The biggest difference between our research and Liang’s was the improvement trend seen in the high kinesiophobia group. The fact that the study population is different is one potential explanation. An additional reason is that, due to the characteristics of the illness, our study has a higher proportion of female participants than male participants. This suggests that the kinesiophobia trajectories of different diseases may have common views as well as their uniqueness.

Baseline factors were significant in differentiating kinesiophobia trajectories. Patients in the rapid recovering group were more likely to have a BMI of 18.5–24.9 compared to the low stable group. That is the level of kinesiophobia in patients whose BMI with normal ranges will be rapidly decreased over time, even if the initial level of kinesiophobia is high. Kocyigit & Akaltun also reported an association between obesity and kinesiophobia in fibromyalgia syndrome [30]. One likely reason was that kinesiophobia in obese patients was primarily related to the somatic focus component of the TSK scale [31]. Moreover, patients following the moderate stable group were more unemployed or retired than those in the low stable group. It is possibly because the employed patients have more social support from their colleagues to cope with kinesiophobia [32]. However, it may not be a coincidence that patients in the slow recovering group were with a high probability of heart disease. Previous research has confirmed that kinesiophobia can be expected in patients with heart problems [33]. Patients who reported moderate or severe pain were more likely to have a high level of kinesiophobia at T0, consistent with the fear-avoidance model. This model suggested that pain was influenced by catastrophizing contributed to kinesiophobia [9, 10]. Although both the rapid recovering group and slow recovering group showed an improvement trend of kinesiophobia, the level of kinesiophobia was higher than that of the low stable group at baseline. This explains why people with moderate-to-severe pain were more likely to be in the slow or rapid recovering group than the low stable group. An interesting aspect of our result is that the odds ratio of pain degree is higher in the rapid recovering group than in the slow recovering group. The possible reason is that patients with moderate-to-severe pain at baseline may receive more support and care from healthcare providers compared with mild pain. To some extent, it promoted the speed of the improvement of fear of movement. In addition, higher odds ratio in rapid recovering group is an indication that the relationship between pain degree and kinesiophobia may be more complex. In the future, longitudinal studies can be conducted to investigate the influence of pain trajectory on the kinesiophobia trajectory.

This research also has implications for the relationship between kinesiophobia trajectories and rehabilitation outcomes. Rehabilitation outcomes were different depending on the kinesiophobia trajectories. As expected, patients in the slow recovering group reported the most psychological distress and the poorest knee function, which remained at a relatively high level of kinesiophobia with only a small-sized decrease from T0 and T2. Meanwhile, patients in this group are worthy of a particular focus because they recover poorly after TKA and may stand to gain the most from psychological rehabilitation. The low stable group consisted of the largest number of patients. These patients showed rather stable and low levels of kinesiophobia from *T*0 to *T*2, thus getting the best social participation level compared to other groups. On the one hand, the other three groups with a high level of kinesiophobia have been associated with physical inactivity and chronic pain development [11], which limits social participation. On the other hand, patients in the low stable group get the optimal psychological state after TKA; thus, they may have a big social network and frequent social connections with family and friends, which facilitate to social participation [32, 34]. Meanwhile, regarding the activities of daily living, patients in all kinesiophobia trajectories experienced good recovery at 3 months after TKA, and no difference was found among the four kinesiophobia trajectories. Furthermore, no differences were observed in activities of daily living between multidisciplinary rehabilitation and usual care in a randomized clinical trial among patients after TKA [1]. Therefore, this finding highlights that TKA may be a significant positive procedure and plays an important role in improving activities of daily living.

### Clinical implications

Clinicians and nurses focused on kinesiophobia after TKA among patients who were obese, unemployed or retired, with heart disease, or with moderate-to-severe pain. As a modifiable factor, early alleviation from kinesiophobia is feasible by providing more targeted education, progressive muscle relaxation, cognitive-behavioral activities, and exercise therapy. In addition, a multidisciplinary team should consider how to assist patients after TKA to improve their rehabilitation outcomes over the kinesiophobia trajectory. However, patients after TKA of the slow recovering group experienced relatively poor rehabilitation outcomes. Therefore, explaining to such patients how to modify mistaken fears and enhancing their positive attitude toward the rehabilitation exercises is crucial to promote comprehensive rehabilitation.

### Study limitations

There were some limitations to take into account when interpreting the results. First, the representativeness of the sample was limited because it was drawn from a single medical institution; thus, the generalizability of the study’s findings was restricted. Moreover, although we corrected the* p* value using the Bonferroni correction in post hoc tests, inflation of type 1 error caused by multiple comparisons cannot be completely ruled out. Third, all variables in this study were derived from self-reported questionnaires, which raised the possibility of common method bias. Fourth, we excluded participants who experienced an adverse event because those patients were readmitted for treatment and supportive medical interventions, which partly excluded patients with poorer rehabilitation outcomes.

## Conclusions

Patients undergoing TKA are vulnerable to a long-lasting kinesiophobia. In the current study, data-driven techniques suggested the existence of four kinesiophobia trajectories among the patients after TKA and the level of kinesiophobia remained fairly stable or improved over time. However, more efforts should be made to identify patients who were at high risk for the slow recovery of kinesiophobia, and interventions need to be customized based on the patients’ needs after TKA depending on each kinesiophobia trajectory.

## Data Availability

The datasets generated and/or analyzed during the current study are not publicly available due to privacy of participants. The data that support the findings of this study are available from the corresponding author, upon reasonable request.

## Abbreviations

TKA: Total knee arthroplasty
TSK: Tampa Scale for Kinesiophobia
K-10: Kessler Psychological Distress Scale
HSS-KS: The Hospital for Special Surgery-Knee Scale
BI: Barthel Index
IPA: The Impact on Participation and Autonomy questionnaire
BMI: Body mass index
ICF: The International Classification of Functioning, Disability and Health
LCGA: Latent class growth analysis
AIC: Akaike information criterion
BIC: Bayesian information criterion
aBIC: Adjusted Bayesian information criterion
VLMR: Vuong–Lo–Mendell–Rubin likelihood ratio test
BLRT: Bootstrapped likelihood ratio test
